# Clinical outcomes of temocillin use for invasive Enterobacterales infections: a single-centre retrospective analysis

**DOI:** 10.1093/jacamr/dlab005

**Published:** 2021-02-14

**Authors:** Katie L Heard, Kieran Killington, Nabeela Mughal, Luke S P Moore, Stephen Hughes

**Affiliations:** 1 Chelsea and Westminster NHS Foundation Trust, 369 Fulham Road, London SW10 9NH, UK; 2 North West London Pathology, Imperial College Healthcare NHS Trust, Fulham Palace Road. London W6 8RF, UK; 3 National Institute for Health Research Health Protection Research Unit in Healthcare Associated Infections and Antimicrobial Resistance, Imperial College London, Hammersmith Campus, Du Cane Road, London W12 0NN, UK

## Abstract

**Background:**

With increasing frequency of resistant Gram-negative bacteria, temocillin has potential utility in reducing carbapenem use. The 2020 EUCAST guideline changes temocillin breakpoints and reclassifies isolates with an MIC of 0.001–16 mg/L as ‘susceptible, increased exposure’ necessitating 6 g/day rather than the previous 4 g/day, associated with significant cost implications.

**Objectives:**

We explore the clinical utility and treatment failure rate of temocillin at 4 g/day dosing.

**Methods:**

All adult inpatient electronic prescriptions of temocillin (3 days or greater) from March 2016 to October 2019 were retrieved using a clinical decision support system (ICNET^®^). Treatment success was defined as survival, no switch to broad-spectrum agent for the same indication and no subsequent recrudescence of infection, occurring within 30 days.

**Results:**

Temocillin was used in 205 eligible patient-episodes, median age 79 years (IQR : 71–87 years), 42.4% female. Median temocillin course length was 5.9 days (IQR : 4.6–7.8 days). Indications for use: urinary tract infection (UTI) (*n *=* *141), pneumonia (*n *=* *53), other (*n *=* *11). In total, 144 (70.2%) patients had targeted treatment; 74 (36.1%) against *Escherichia coli*, 70 (34.4%) other Enterobacterales. A total of 130 (63%) patients received 4 g/day; the remaining patients had reduced renal function with dosing in accordance with guidance. Overall temocillin treatment success was 79.5%; highest when used to treat UTI 85.8% (versus 67.9% in respiratory infections, *P *=* *0.008). Empirical treatment demonstrated 82.0% (50/61) success [versus 78.5% (113/144) among targeted treatment, *P *=* *0. 71].

**Conclusions:**

Temocillin at 4 g/day is an effective and safe alternative in treating patients with Gram-negative infections, but should be considered in the context of patient age and comorbidities. Increased dosing or alternate strategies may be indicated when the infection is not of a urinary source.

## Introduction

Enterobacterales commonly cause serious community-acquired and healthcare-associated infections. ESBLs are major causes of β-lactam (penicillin and cephalosporin) resistance in Enterobacterales. ESBLs are often expressed with other genes that confer fluoroquinolone and aminoglycoside resistance, further reducing treatment options. ESBLs are a growing problem globally, with dramatic increases in these resistant organisms occurring in both community and hospital settings.^1^ As a result, carbapenems are often used as treatment options for ESBL-producing Enterobacterales, but resistance to carbapenems among Gram-negative bacteria has also increased over the last decade. Outbreaks of carbapenem-resistant Enterobacterales (CRE) are associated with high costs and high mortality.^2^ It is therefore essential to consider non-carbapenem antimicrobial agents where possible.

Temocillin, a 6-alpha-methoxy derivative of ticarcillin, is a narrow spectrum β-lactam with activity against many Gram-negative bacteria, but not Gram-positive bacteria, anaerobes or *Pseudomonas aeruginosa*.^3^ It is licensed in the UK for the treatment urinary tract infections (UTIs) and lower respiratory tract infections (LRTIs) where susceptible Enterobacterales are suspected or confirmed.^4^ Temocillin is licensed at doses of 4–6 g/day (in 2–3 divided doses or via continuous infusion).^5^ A 2011 review of temocillin highlighted that the modal MIC for Enterobacterales was 4–8 mg/L (range 2–32 mg/L) with 90% of isolates susceptible at 16 mg/L, and that temocillin is stable against a variety of β-lactamases, including ESBLs (TEM, SHV, CTX-M), AmpC β-lactamases and KPC carbapenemases.^6^^,^^7^ Where susceptible, temocillin offers a useful alternative to carbapenems.

In 2020, EUCAST published clinical breakpoints for temocillin based on WT distribution, reducing the breakpoint for susceptible isolates to <0.001 mg/L. Those with an MIC of 0.001–16 mg/L are reclassified as ‘susceptible, increased exposure’ (I) and high-dose temocillin regimens are recommended (6 g/day).^8^ Previously BSAC guidance advised a breakpoint of 8 mg/L for systemic infection (or 32 mg/L for urine infections),^9^ which should be overcome by 4 g/day temocillin.

Implementation of the 2020 EUCAST breakpoint would necessitate high-dose temocillin (6 g/day) for the majority of clinical cases. This has significant financial impact on healthcare providers and may cause many centres in the UK to revert to low-cost carbapenems, driving further development of CRE. To understand current clinical utility at 4 g/day dosing, we undertook a single-centre retrospective observational analysis of the outcomes of patients prescribed temocillin.

At the study centre temocillin is included within the guidelines as empirical therapy for the treatment of UTIs; when the patient has a history of ESBL organisms susceptible to temocillin or is at high risk of such organisms. Other use is based on targeted therapy once a causative organism resistant to first line agents is known or based on the advice of the antimicrobial stewardship team when empirical therapy is failing.

## Methods

### Study setting and design

A retrospective observational analysis was undertaken of all hospitalized patients treated with temocillin for 3 or more days across a large single-centre NHS acute Trust; Chelsea and Westminster NHS hospital (London, UK). All adults (>18 years) treated with temocillin between 26 March 2016 and 31 October 2019 were included. Temocillin was prescribed for patients with known susceptible organisms where other first line agents were not suitable (i.e. in the presence of ESBL- or AmpC-producing organisms) or if there was a high suspicion of an ESBL infection by infection specialists. Temocillin was prescribed at reduced dosing as per local prescribing policy in patients with renal dysfunction.

### Data extraction and analysis

A clinical decision support system (ICNET^®^, Baxter, UK) was used as previously described^10^ to identify patients treated with temocillin. This included microbiology culture and susceptibility data as well as patient demographic and clinical data, including mortality, renal function, readmission, prior antimicrobials, intensive care episodes, antimicrobial escalation and cases of *Clostridioides difficile*. Data were anonymized at the point of collection, tabulated, and descriptive statistics undertaken using SPSS Statistics (Version 26, IBM Corp., Armonk, NY, USA).

### Definitions

Treatment success was defined as all of: patient survival, no antibiotic escalation for the same indication [escalation was classified as a change of spectrum either to an antipseudomonal agent (ceftazidime or piperacillin/tazobactam) or carbapenem (ertapenem or meropenem)] and no subsequent treatment for the same infection (all within 30 days). As a secondary outcome patients’ *C. difficile* status was reviewed at 30 days. Patients changed from temocillin to an oral treatment or a once-daily administered IV antimicrobial [to enable outpatient parental antimicrobial therapy (OPAT)] were not defined as a treatment failure. Patients who changed treatment due to a change of infection source or new microbiology were excluded.

Temocillin use was considered empirical if the patient had no clinically relevant microbiology results in the past 30 days. Only the first treatment course of temocillin on each patient admission or within 30 days was included for patients receiving multiple courses of temocillin. Patients who received 6 g thrice daily or renal equivalent were excluded. Temocillin was dosed at 2 g twice daily for CL_CR_ >40 mL/min, 1 g twice daily with 2 g immediate dose for CL_CR_ 20–40 mL/min and 1 g once daily with 2 g immediate dose for CL_CR_ <20 mL/min.

Enterobacterales were speciated and grouped as inhibitor-resistant TEM (IRT), AmpC, ESBL or carbapenem-resistant Enterobacterales (CRE) based on resistance patterns; to prevent duplication only the most resistant mechanism (IRT<AmpC<ESBL<CRE) was counted in the results analysis. Renal function was estimated using eGFR (Modification of Diet in Renal Disease) to ensure adequate dosing as per trust guidelines.

Antibiotics received in the 7 days prior to temocillin were reviewed and analysed. Antibiotics were classified into aminoglycoside, carbapenem and other β-lactam. Duration was grouped by less than 72 h received and greater than 72 h.

Intensive care treatment in the 7 days pre or post temocillin initiation was reviewed in order to consider patients who may be deemed more unwell or be receiving renal replacement therapy.

Hospital-acquired pneumonia was defined as pneumonia onset >2 days after admission or within 28 days of a previous admission.

### Study approval

The project was registered and approved by the Chelsea and Westminster NHS Foundation Trust clinical governance department (reference AP_86) and the need for individual consent was waived for this retrospective analysis following review by the Chelsea and Westminster NHS Foundation Trust Research & Governance Office. All data were collected and stored in concordance with the Data Protection Act and the General Data Protection Regulation.

### Availability of data and materials

The dataset reported within this study are available from the first author (K.L.H., katie.heard@nhs.net) on reasonable request, as long as this meets local ethics and research governance criteria.

## Results

### Patient cohort

**Figure 1.**
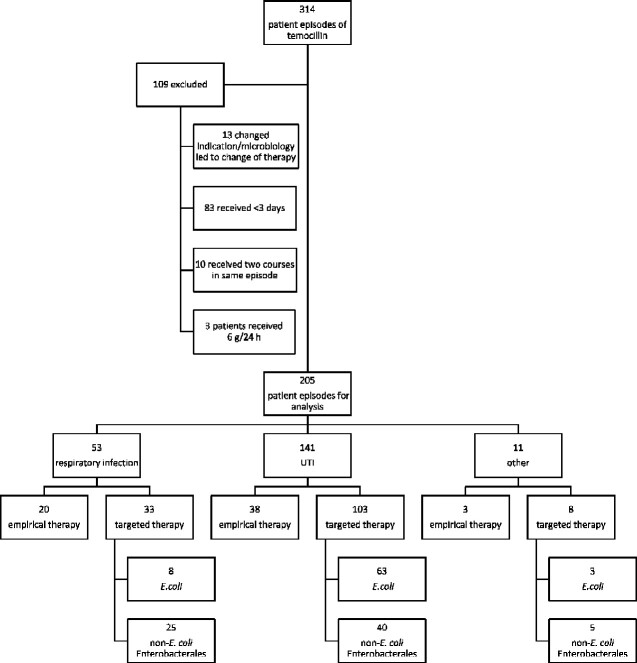
Clinical utility of temocillin, London, 2016–19; inclusion and exclusion criteria and cohort characteristics.

During the study period 314 temocillin treatment courses for 280 patients were identified. In total, 109 treatment courses were excluded from final analysis (83 were treated for <3 days, 10 patients received multiple courses in one episode, 3 patients received 2 g q8h and 13 patients required treatment alteration due to revision of infection source or microbiology after 3 days). A total of 205 courses met study inclusion criteria and were included in final analysis (Figure 1). The median age was 79 years (IQR 71–87 years), 87/205 (42.4%) were female. Four patients received treatment in intensive care within 7 days of initiating temocillin. Temocillin was initiated on median of day 4.1 of admission (IQR 1.5–12.5 days) and median total length of stay was 19.5 days (IQR 10.5–42.6 days). Historic *C. difficile* carriage was identified in 10/205 (4.9%) patients at the time of temocillin initiation. The median temocillin course length was 5.9 days (IQR 4.6–7.8 days).

In total, 141 (68.8%) patients were treated for urinary infections, 53 (25.9%) for chest and 11 (5.4%) other indications, including cellulitis (*n *= 3), unknown source (*n *= 2), abdomen (*n *= 3), prostate (*n *= 2) and peri-anal abscess (*n *= 1) (Table 1). Temocillin was dosed at 4 g/day (2 g IV q12h), except in cases of reduced renal function where dosing followed local guidance. Six patients received doses lower than suggested in guidelines, none of whom failed therapy.

**Table 1.** Clinical characteristics of patients receiving temocillin, London, 2016–19

	Urine, *n *=* *141	Chest, *n *=* *53	Other^a^, *n *=* *11	Total, *n *=* *205
Age, years, median (IQR)	81 (71–88)	77 (71–83)	74 (71–87)	79 (71–87)
Male, *n* (%)	81 (57.4)	33 (62.3)	4 (36.3)	118 (57.6)
Renal function, eGFR mL/min, median (IQR)	59 (34–87)	84 (57.5–90)	65 (34.5–81)	66.5(35.3–90)
LoS pre-temocillin, days, median (IQR)	2.6 (1.0–10.8)	8.38 (3.5–12.7)	5.6 (2.6-13.3)	4.1 (1.5–12.5)
Total LoS, days, median (IQR)	18.9 (9.4–42.9)	20.3 (12.7–39.8)	32.0 (17.9–39.9)	19.5 (10.5–42.6)
Previous CDI positive, *n*, by PCR (by toxin)	8 (1)	2 (1)	0	10 (2)

LoS, length of stay; CDI, *C. difficile* infection.

a Other: prostatitis, skin and soft tissue, pyrexia of unknown origin, abdominal infection.

### Treatment success

Overall temocillin treatment success was 79.5% (163/205), varying by treatment indication and by causative organism (Table 2). Success was highest when used to treat UTI at 85.8% (121/141) compared with LRTIs at 67.9% (36/53; *P *=* *0.008). Other indications for temocillin use were heterogeneous in nature and success was just 54.5% (6/11). Temocillin therapy was targeted against known susceptible pathogens in 70.2% (144/205) episodes, of which 78.5% (113/144) were successful, compared with 82.0% successful outcomes in those prescribed temocillin in culture-negative infection (50/61; *P *=* *0.76). Patients treated for bacterial infections with known resistance mechanisms (ESBL, AmpC, IRT, CRE) had similar success rates to those with WT bacterial/culture negative infections, 77.7% (94/121) versus 82.1% (69/84) respectively, *P *=* *0.55. For further sub-analysis see Table 2.

**Table 2.** Outcome data of patients receiving temocillin, London, 2016–19

	Urine, *n *=* *141	Chest, *n *=* *53	Other^a^, *n *=* *11	Total, *n *=* *205
Temocillin treatment, days, median (IQR)	5.9 (4.5–7.8)	6.0 (5.0–7.8)	6.0 (5.7–8.2)	5.9 (4.6–7.8)
Dose, g, median (IQR)	4 (2–4)	4 (4–4)	4 (2–4)	4 (2–4)
Treatment success at 30 days, *n* (%)	121(85.8)	36 (67.9)	6 (54.5)	163 (79.5)
Targeted therapy, *n* (%)	103 (73.0)	33 (62.3)	8 (72.7)	144 (70.2)
success, *n* (%)	86 (83.5)	22 (66.7)	5 (62.5)	113 (78.5)
Blood culture positive, *n* (%)	34 (24.1)	3 (5.7)	3 (27.2)	40 (19.5)
success, *n* (%)	29 (85.3)	2 (66.7)	3 (100.0)	34 (85.0)
Culture negative, *n* (%)	38 (27.0)	20 (37.7)	3 (27.3)	61 (29.8)
success, *n* (%)	35 (92.1)	14 (70.0)	1 (33.3)	50 (82.0)
*E. coli*, *n* (%)	63 (44.7)	8 (15.1)	3(27.3)	74 (36.1)
* *WT, *n* (%)	9 (14.3)	0 (0)	1 (33.3)	10 (13.5)
success, *n* (%)	7 (87.4)	—	0 (0.0)	7 (70.0)
IRT, *n* (%)	2 (3.2)	1 (12.5)	1 (33.3)	4 (5.4)
success, *n* (%)	2 (100.0)	1 (100.0)	0 (0.0)	3 (75.0)
ESBL, *n* (%)	52 (82.6)	7 (87.5)	1 (33.3)	60 (81.1)
success, *n* (%)	45 (86.5)	5 (71.4)	1 (100.0)	51 (85.0)
CRE, *n* (%)	0 (0)	0 (0)	0 (0)	0 (0)
Non-*E. coli* Enterobacterales, *n* (%)	40 (28.4)	25 (47.2)	5 (45.5)	70 (34.1)
* *WT^b^, *n* (%)	10 (25.0)	3 (12.0)	0 (0)	13 (18.5)
success, *n* (%)	9 (90.0)	3 (100.0)	—	12 (92.3)
AmpC, *n* (%)	10 (25.0)	12 (48.0)	4 (80.0)	26 (41.4)
success, *n* (%)	8 (80.0)	8 (66.7)	4 (100.0)	20 (76.9)
ESBL, *n* (%)	18 (45.0)	10 (40.0)	1 (20.0)	29 (41.4)
success, *n* (%)	14 (77.8)	5 (50%)	1 (100.0)	20 (69.0)
CRE *n* (%)	2 (5.0)	0 (0)	0 (0)	2 (2.9)
success, *n* (%)	1 (50.0)	—	—	1 (50.0)
Treatment failure, 30 days, *n* (%)	20 (14.1)	17 (32.1)	5 (45.5)	42 (20.5)
reinfection, *n* (%)	8 (40.0)	6 (35.3)	1 (20.0)	15 (35.7)
in-hospital mortality, *n* (%)	5 (25.0)	9 (52.9)	1 (20.0)	15 (35.7)
change of therapy (same indication), *n* (%)	7 (35.0)	2 (11.8)	3 (60.0)	12 (28.6)
carbapenem, *n* (%)	4 (57.1)	1 (50.0)	3 (100.0)	8 (66.6)
antipseudomonal, *n* (%)	3 (42.9)	1 (50.0)	0 (0.0)	4 (33.3)
Secondary outcome: *C. difficile* infection at 30 days, *n*	0	0	0	0

AmpC, AmpC β-lactamases; IRT, inhibitor-resistant TEM; CRE, carbapenem-resistant Enterobacterales.

a Other, prostatitis, skin and soft tissue, pyrexia of unknown origin, abdominal infection.

b No identified resistance mechanism.

Among those patients with UTIs, per-pathogen analysis demonstrated treatment success was 85.7% (54/63), 80% (32/40) and 92.1% (35/38) for patients with *E. coli*, non-*E.coli* Enterobacterales and empirical therapy, respectively . 24.1% (34/141) of patients treated for urinary infection has a concurrent bacteraemia identified; the presence or absence of associated bacteraemia had no significant impact on treatment success (85.3% versus 85.9%; *P *=* *0.86).

Among those patients with LRTIs, per-pathogen analysis demonstrated treatment success was 75% (6/8), 64.0% (16/25) and 70.0% (14/20) for treatment of *E. coli*, non *E. coli* Enterobacterales, and empirical therapy, respectively. Only 3/53 (5.7%) patients treated for LRTI had a concurrent bacteraemia; the presence or absence of associated bacteraemia had no significant impact on outcome (success rate 68.0% versus 66.7%, *P *=* *1). 88.7% (47/53) patients were treated for a hospital acquired pneumonia. One patient with LRTI had recent mechanical ventilation exposure. Temocillin was not routinely combined with anti-Gram positive therapy (only 3/58 cases).

#### Prior antibiotic therapy

A total of 129 (62.9%) patients received one or more antibiotic prescriptions in the 7 days preceding temocillin initiation. This was commonly an aminoglycoside (85/129) administered in patients for less than 72 h (78/85) typically for UTI indication where amikacin is used for empirical therapy for the first 24 h. Carbapenem exposure prior to temocillin was low (two patients treated for UTI exposed to meropenem); non-ESBL targeting β-lactams were used in 43 episodes with 16/43 receiving more than 72 h treatment. Use of antibiotics prior to temocillin did not impact on treatment outcomes (78.3% versus 82.9% treatment success in the patients receiving antibiotics before temocillin and not receiving antibiotics, respectively). Use of an aminoglycoside, carbapenem or other β-lactams did not impact on treatment success rates.

#### Intensive care stay

Within this cohort study, four patients received temocillin treatment were managed in the intensive care unit at the time initiation. All four patients had successful outcomes.

### Treatment failure

A total of 42 (20.4%) treatment episodes had a study defined treatment failure, and are explored below.

### Death

Of those who received temocillin, 7.3% (15/205) died within 30 days of treatment (Table 2), median age 79 years (IQR: 72.5–89 years), this was no different to the survival group (*P *=* *0.53). Of the patients who died, 26.7% (4/15) had treatment empirically changed with no new microbiology prior to death; two patients were changed to meropenem and two received antipseudomonal β-lactams.

### Escalation of therapy

Of patients who were initiated on temocillin, 5.9% (12/205) changed therapy due to non-improvement, or deterioration, in clinical parameters (Table 2). Three patients who were escalated were being treated with temocillin for a non-UTI, non-LRTI indication.

### Reinfection

In total, 7.3% (15/205) of patients were treated for an infection in the same organ system in the 30 days after completing temocillin. Eight patients had been discharged and readmitted and seven were treated within the same episode. Seven patients were treated for recurrent UTIs, three of whom were known to suffer frequent UTIs, one who had ureteric stents. Six patients were retreated for chest infections. One patient with an abdominal infection went back on temocillin and subsequently achieved success, the failure was likely due to source control and duration of therapy.

### Adverse events

#### C. difficile

No patients were positive for *C. difficile* within 30 days post-completion of temocillin therapy, including those noted to have *C. difficile* prior to temocillin therapy. One patient had episodes of *C. difficile* negative (PCR negative and toxin negative) diarrhoea. There were no incidences of other severe adverse effects.

### Utility as a carbapenem sparing agent

Over 1250 days of temocillin therapy were observed across the study. This use was in patients for whom carbapenems may otherwise have been considered.

## Discussion

We find nearly an 80% clinical success rate with temocillin for the treatment of invasive bacterial infection. A high burden of MDR bacteria was identified within the study population and temocillin provided a valuable alternative to carbapenems. In response to increasing antimicrobial resistance, the availability of effective non-carbapenem therapies for the treatment of invasive ESBL and CRE infections is increasingly important.

Our rate of clinical success in respiratory infections is comparable to the literature, with clinical cure rates of 70% generally evident in Phase III studies of nosocomial infection using other agents such as ceftazidime-avibactam and meropenem.^11^ For temocillin, the lack of activity against *P. aeruginosa* limits its role in pneumonia, particularly in ventilator-associated infection where pseudomonal infections are common. In the absence of *Pseudomonas* spp. or for directed therapy for Enterobacterales infection, we find temocillin provides a feasible option for treatment of pneumonia in the presence of MDR phenotypes. Gram-positive coverage, if indicated, can be covered by the addition of appropriate other therapy (we used flucloxacillin or linezolid).

Our rate of success in UTI is in line with the more established role of temocillin in the management of complicated urinary infections where MDR Gram-negatives are more prevalent. Monotherapy temocillin provided robust treatment with clinical success exceeding 80%. A high rate of concurrent bacteraemia infections was identified but we found no negative impact on treatment outcomes among that subset of patients. A dose of 4 g/day was advised as default, with dose adjustments for renal failure. This high treatment success is comparable to published studies of a variety of agents used to treat MDR UTIs (including fosfomycin, imipenem/cilastatin, cefiderocol and ceftazidime/avibactam).^12–15^

**Figure 2.**
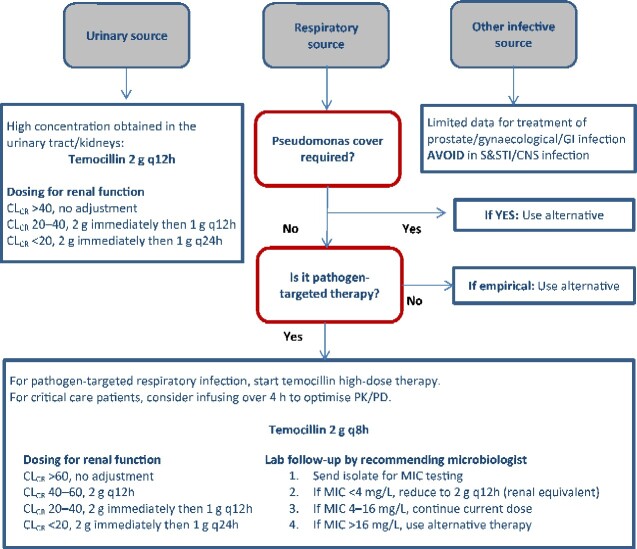
Proposed clinical algorithm for in patient use of temocillin, London, 2020. PK/PD, pharmacokinetics/pharmacodynamics; S&STI, skin and soft tissue infection; GI, gastrointestinal.

The optimum dosing of temocillin for treatment of MDR infections cannot be defined from this retrospective analysis due to our lack of comparator with high-dose 6 g/day dosing. However, our success rates with 4 g/day have meant our dosing strategies have not been routinely adjusted to 6 g/day following licensing revision in 2018^8^ or the EUCAST change in breakpoint in 2020.^16^ The MIC is not routinely requested but is derived however for deep-seated or CRE infections to enable patient-tailored dosing, and MIC values of 8–16 mg/L are treated with 6 g/day. In patients with renal dysfunction, a local guideline with doses exceeding the manufacture’s guidance has been developed. All patients receive a 2 g loading dose irrespective of renal function and dose adjustments for CL_CR_ ≥40 mL/min are not undertaken. Compositing all these aspects of our data, we propose a tailored treatment strategy for temocillin (Figure 2).

Our analysis has several limitations. First, this real-world retrospective study was completed with no control group due to the heterogeneity of clinical presentation and microbiology history of these patients. We were unable to match patients to a suitable control group (e.g. carbapenem) within our study due to these many variables; a prospective randomized control trial would be needed to confirm non-inferiority of temocillin to other anti-ESBL therapies. We recommend that such future studies are completed to confirm the assumptions of our work. Second, the retrospective nature of this study may affect the fidelity of the data as they were collated from clinical notes rather than being collected prospectively for the purposes of a trial. Third, we have very few patients dosed at 6 g/day to act as a comparator; future work should be done comparing the outcomes of both dosing schedules, and in particular looking for the value added (or any increase in adverse events) from the higher dose. Fourth, we did not look at source control or the presence of hardware e.g. urinary stents; a deeper review to include such data may explain some of the failures experienced. Finally, whilst our dataset focuses on clinical outcomes of patients treated with temocillin, our data do not include the financial implications of its use as a carbapenem-sparing agent. A separate analysis of the financial implications of the use of temocillin (at either 4 g/day or 6 g/day) and other antimicrobials for MDR organisms is urgently needed, yet this must be undertaken from a whole-healthcare economy perspective or cheaper yet broader-spectrum agents may be misconstrued.

In conclusion, we find temocillin offers a narrow spectrum option for the treatment of resistant Gram-negative infections. The EUCAST breakpoint (2020)^16^ recommendations advising dosing at 6 g/day to cover pathogens with MIC 0.001–16 mg/L instigated this retrospective analysis, yet we find dosing at (the previously licensed) 4 g/day demonstrates favourable outcomes for the majority of infections, particularly UTI, where accumulation of active temocillin occurs. For complex respiratory infections, where temocillin tissue concentration is reduced, high-dose (6 g/day) therapy may be indicated, and possibly extended interval (4 h infusion) administration may have utility; specific further work in this cohort is warranted. Judicious use of temocillin dosing enables the balancing of antimicrobial stewardship goals against budget constraints to maintain total medication spend.
